# Bioinformatics clouds for big data manipulation

**DOI:** 10.1186/1745-6150-7-43

**Published:** 2012-11-28

**Authors:** Lin Dai, Xin Gao, Yan Guo, Jingfa Xiao, Zhang Zhang

**Affiliations:** 1 CAS Key Laboratory of Genome Sciences and Information, Beijing Institute of Genomics, Chinese Academy of Sciences, No.7 Beitucheng West Road, Building G, Chaoyang District, Beijing, 100029, China; 2 School of Computer Science and Technology, Beijing Institute of Technology, Beijing, 100081, China; 3 Computer, Electrical and Mathematical Sciences and Engineering Division, King Abdullah University of Science and Technology (KAUST), Thuwal, 23955-6900, Saudi Arabia; 4 Cloud Development and Cloud Solution Integration, IBM China Systems & Technology Lab, IBM Co. Ltd, Beijing, 100193, China

**Keywords:** Cloud computing, Bioinformatics, Big data, Data storage, Data analysis

## Abstract

**Abstract:**

As advances in life sciences and information technology bring profound influences on bioinformatics due to its interdisciplinary nature, bioinformatics is experiencing a new leap-forward from in-house computing infrastructure into utility-supplied cloud computing delivered over the Internet, in order to handle the vast quantities of biological data generated by high-throughput experimental technologies. Albeit relatively new, cloud computing promises to address big data storage and analysis issues in the bioinformatics field. Here we review extant cloud-based services in bioinformatics, classify them into Data as a Service (DaaS), Software as a Service (SaaS), Platform as a Service (PaaS), and Infrastructure as a Service (IaaS), and present our perspectives on the adoption of cloud computing in bioinformatics.

**Reviewers:**

This article was reviewed by Frank Eisenhaber, Igor Zhulin, and Sandor Pongor.

## Background

With significant advances in high-throughput sequencing technologies and consequently the exponential expansion of biological data, bioinformatics encounters difficulties in storage and analysis of vast amounts of biological data. The gap between sequencing throughput and computer capabilities in dealing with such big data is growing [1]. As reported on February 2012, two nanopore sequencing platforms (GridION and MinION) are capable of delivering ultra-long sequencing reads (~100kb) with additionally higher throughput and much lower cost [2]. This means that biological data will accumulate at an ever-faster pace. Digging out the “treasure” from massive biological data represents the primary challenge in bioinformatics, consequently placing unprecedented demands on big data storage and analysis. With the amount of data growing continuously, it is becoming increasingly daunting for small laboratories or even large institutions to establish and maintain computational infrastructures for data processing. At present, a promising solution to address this challenge is cloud computing [3-5], which exploits the full potential of multiple computers and delivers computation and storage as dynamically allocated virtual resources via the Internet [6].

## Review

### Cloud computing as a public utility

The term of “cloud computing” was inspired by the cloud symbol that is often employed to depict the Internet in flowcharts. As a matter of fact, cloud computing is not a new concept; it can date back to 1961 at the MIT Centennial when John McCarthy opined that “computation may someday be organized as a public utility” [7]. Cloud computing makes the best use of multiple computers to provide convenient and on-demand access to hosted resources (e.g., computation, storage, applications, servers, network) via Web Application Programming Interfaces (API). Due to its efficient and economical features, it is believed that cloud computing gains promise in transforming computing into a public utility [8]. Similar to extant public utilities (viz., water, electricity, gas, and telephone), computing utility packages a variety of computer resources as metered services (“*pay-as-you-go*”), which can be accessed by any person without the necessity to know where the services are hosted or how they are delivered. Whether a public utility or not, cloud computing has already become a significant technology in big data storage and analysis, exerting revolutionary influences on both academia and industry.

### Cloud-based resources in bioinformatics

**Figure 1:**
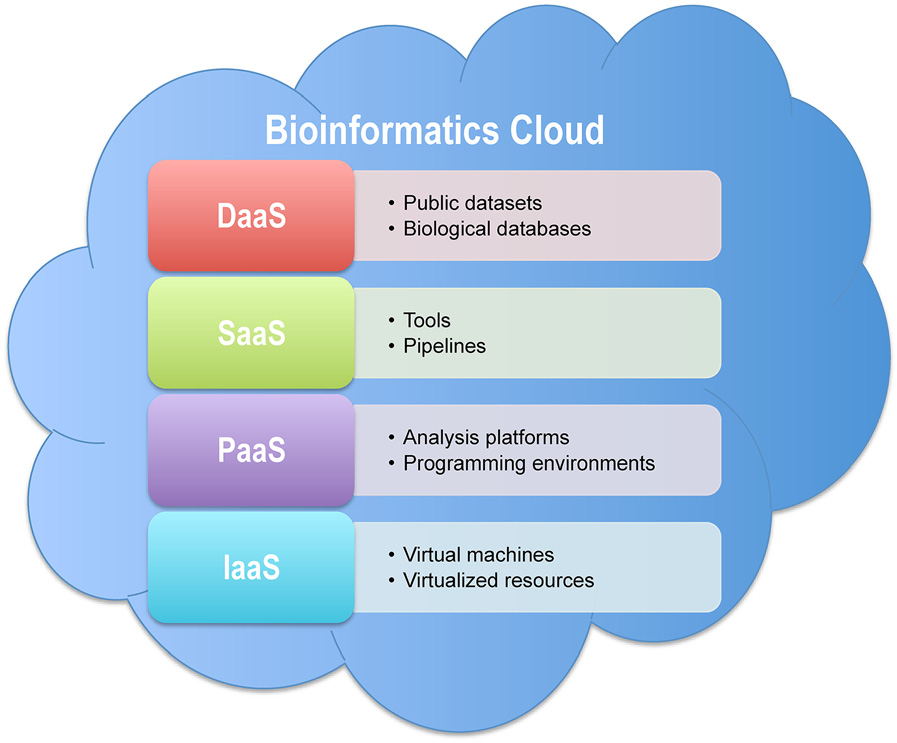
**Illustration of bioinformatics cloud.** Cloud-based services in bioinformatics are grouped into Data as a Service (DaaS), Software as a Service (SaaS), Platform as a Service (PaaS), and Infrastructure as a Service (IaaS).

The popularization of cloud computing is to some extent attributable to open source software in aid of its implementation, such as Hadoop and associated software. Hadoop (http://hadoop.apache.org) features two key modules—MapReduce and Hadoop Distributed File System (HDFS). MapReduce divides a computational program into many small sub-problems and distributes them on multiple computer nodes, and HDFS provides a distributed file system that stores data on these nodes. Hadoop and its associated software are designed to handle load balancing among multiple nodes and to detect node failures that can be automatically re-executed on any node. In brief, Hadoop allows for the distributed processing of large datasets across multiple computer nodes, supports big data scaling (HDFS, HBase), and enables fault-tolerant parallelized analysis (MapReduce). Thus, Hadoop meets the needs of bioinformatics and several studies have successfully used Hadoop in bioinformatics [9-11], accordingly leading to cloud-based bioinformatics resources. As mentioned, cloud computing delivers hosted services over the Internet. Thus, bioinformatics clouds involve a large variety of services from data storage, data acquisition, to data analysis, which in general fall into four categories (Figure 1), viz., Data as a Service, Software as a Service, Platform as a Service, and Infrastructure as a Service [12]. In what follows, we summarize existing cloud-based resources in bioinformatics and classify them into these four categories (Table 1).

**Table 1:** Cloud resources in bioinformatics

**Resource**	**Description & availability**
**Data as a Service (DaaS):**	
AWS Public Datasets	Cloud-based archives of GenBank, Ensembl, 1000 Genomes, Model Organism Encyclopedia of DNA Elements, Unigene, Influenza Virus, etc.; http://aws.amazon.com/publicdatasets
**Software as a Service (SaaS):**	
BGI Cloud (unpublished)	Cloud-based implementations of various genomic analysis applications; http://cloud.genomics.cn
CloudAligner [16]	Fast and full-featured MapReduce-based tool for sequence mapping; http://cloudaligner.sourceforge.net
CloudBLAST [19]	A cloud-based implementation of NCBI BLAST; http://ammatsun.acis.ufl.edu/amwiki/index.php/CloudBLAST_Project
CloudBurst [17]	Highly sensitive short read mapping with MapReduce; http://cloudburst-bio.sourceforge.net
Contrail (unpublished)	Cloud-based *de novo* assembly of large genomes; http://contrail-bio.sourceforge.net
Crossbow [18]	Read Mapping and SNP calling using cloud computing; http://bowtie-bio.sf.net/crossbow
EasyGenomics (unpublished)	Cloud-based NGS pipelines for whole genome resequencing, exome resequencing, RNA-Seq, small RNA and de novo assembly; http://www.easygenomics.org
eCEO [26]	Cloud-based identification of large-scale epistatic interactions in genome-wide association study (GWAS); http://www.comp.nus.edu.sg/~wangzk/eCEO.html
FX [20]	RNA-Seq analysis tool; http://fx.gmi.ac.kr
Gaea (unpublished)	Cloud-based genome re-sequencing assembly; http://bgiamericas.com/data-analysis/cloud-computing
Hecate (unpublished)	Cloud-based *de novo* assembly; http://bgiamericas.com/data-analysis/cloud-computing
Jnomics (unpublished)	Cloud-scale sequence analysis suite based on Apache Hadoop; http://sourceforge.net/apps/mediawiki/jnomics
Myrna [21]	Differential gene expression tool for RNA-Seq; http://bowtie-bio.sourceforge.net/myrna
PeakRanger [24]	Cloud-enabled peak caller for ChIP-seq data; http://www.modencode.org/software/ranger
RSD [23]	Reciprocal smallest distance algorithm for ortholog detection using Amazon's Elastic Computing Cloud; http://roundup.hms.harvard.edu
VAT [25]	Variant annotation tool to functionally annotate variants from multiple personal genomes at the transcript level; http://vat.gersteinlab.org
YunBe [22]	Pathway-based or gene set analysis of expression data; http://tinyurl.com/yunbedownload
**Platform as a Service (PaaS):**	
Eoulsan [27]	Cloud-based platform for high throughput sequencing analyses; http://transcriptome.ens.fr/eoulsan
Galaxy Cloud [28,29]	Cloud-scale Galaxy for large-scale data analysis; http://galaxy.psu.edu
**Infrastructure as a Service (IaaS):**	
Cloud BioLinux [30]	A publicly accessible virtual machine for high performance bioinformatics computing using cloud platforms; http://cloudbiolinux.org
CloVR [31]	A portable virtual machine for automated sequence analysis using cloud computing; http://clovr.org

#### Data as a service

Bioinformatics clouds are heavily dependent on data, as data are fundamentally crucial for downstream analyses and knowledge discovery. It is reported that annual worldwide sequencing capacity is currently beyond 13 Pbp and still on the increase annually by a factor of five (http://sourceforge.net/apps/mediawiki/jnomics). Due to such unprecedented growth in biological data, delivering Data as a Service (DaaS) via the Internet is of utmost importance [13,14]. DaaS enables dynamic data access on demand and provides up-to-date data that are accessible by a wide range of devices that are connected over the Web. A case in point is Amazon Web Services (AWS) which provides a centralized repository of public data sets, including archives of GenBank, Ensembl, 1000 Genomes, Model Organism Encyclopedia of DNA Elements, Unigene, Influenza Virus, etc. As a matter of fact, AWS contains multiple public datasets for a variety of scientific fields, such as biology, astronomy, chemistry, climate, economics, etc. (http://aws.amazon.com/publicdatasets). All public datasets in AWS are delivered as services and thus can be seamlessly integrated into cloud-based applications [15].

#### Software as a service

Bioinformatics requires a large variety of software tools for different types of data analyses. Software as a Service (SaaS) delivers software services online and facilitates remote access to available bioinformatics software tools through the Internet. As a consequence, SaaS eliminates the need for local installation and eases software maintenances and updates, providing up-to-date cloud-based services for bioinformatic data analysis over the Web. In the past several years, efforts have been made to develop cloud-scale tools, including sequence mapping [16-18], alignment [19], assembly (Contrail, Gaea and Hecate; unpublished), expression analysis [20-22], sequence analysis (Jnomics; unpublished), orthology detection [23], peak caller for ChIP-seq data [24], functional annotation of variants from multiple personal genomes [25], identification of epistatic interactions of single nucleotide polymorphisms (SNPs) [26], and various cloud-based applications for NGS (Next-Generation Sequencing) data analysis (BGI Cloud and EasyGenomics; unpublished).

#### Platform as a service

To make the cloud programmable, Platform as a Service (PaaS) offers an environment for users to develop, test and deploy cloud applications where computer resources scale automatically and dynamically to match application demand, so that users do not need to know how many resources are required or to assign resources manually in advance. PaaS features rapid application development and good scalability, presenting usefulness in developing specific applications for big biological data analysis. Typically, the environment delivered by PaaS includes programming language execution environments, web servers, and databases. From this point, by delivering data as a service and functioning as a database, DaaS can be regarded as an enhancement of PaaS. Currently, there are only two PaaS platforms in bioinformatics belonging to web servers, namely, Eoulsan [27], which is a cloud-based platform for high-throughput sequencing analyses, and Galaxy Cloud [28,29], which is a cloud-scale Galaxy for large-scale data analyses.

#### Infrastructure as a service

To reach the full potential of computer resources, Infrastructure as a service (IaaS) offers a full computer infrastructure by delivering all kinds of virtualized resources via the Internet, including hardware (e.g., CPUs) and software (e.g., operating systems). Users can access virtualized resources as a public utility and pay for the cloud resources that they utilize. Since different users often need different cloud resources, flexibility and customization are essential to IaaS. With the ongoing and rapid advancement of IT, it is increasingly efficient to run applications within Virtual Machines (VMs). VM isolates users from the underlying infrastructure and provides flexibility to meet the customized needs of different users. To date, there are two examples of IaaS in bioinformatics, viz., Cloud BioLinux [30], which is a publicly accessible VM for high-performance bioinformatics computing, and CloVR [31], which is a portable VM that incorporates several pipelines for automated sequence analysis.

### Toward bioinformatics clouds

Albeit relatively new, cloud computing holds great promise in effectively addressing big data storage and analysis problems in bioinformatics. Below, we present our perspectives on the adoption of cloud computing in bioinformatics research.

#### Placing data and software into the cloud

The traditional way for bioinformatics analysis often involves downloading data from public sites (e.g., NCBI, Ensembl), installing software tools locally, and running analyses on in-house computer resources. By placing data and software into the cloud and delivering them as services (namely, DaaS and SaaS), data and software can be seamlessly and easily integrated into the cloud so as to achieve big data storage and analysis. Thus, it would be desirable to store and analyze big biological data within the cloud.

In the era of big data, however, only a tiny amount of biological data is accessible in the cloud at present (only AWS, including GenBank, Ensembl, 1000 Genomes, etc.) and the vast majority of data are still deposited in conventional biological databases. In the long run, more and more sequencing projects, such as the Genome 10K Project (a collection of DNA sequences representing the genomes of 10,000 vertebrate species; http://www.genome10k.org), the 1001 Genomes Project (a catalog of genetic variation in 1001 strains of *Arabidopsis thaliana*; http://www.1001genomes.org), the 1KITE Project (1K Insect Transcriptome Evolution; http://www.1kite.org), TCGA (the Cancer Genome Atlas; http://cancergenome.nih.gov), etc., would generate ultra-large volumes of biological data and thus require bioinformatics clouds for big data storage, sharing and analysis [32]. In addition, to decipher the most important and complex biological questions often involves the utilization of multiple tools [33]. However, extant efforts have only touched a small fraction of cloud-based tools. Most software tools are written for desktop (rather than cloud) [34] and therefore are not provided as cloud-based web services accessible via the Web, making it infeasible to perform complex bioinformatics tasks. To fulfill big data storage, sharing and analysis with lower cost and higher efficiency, it is essentially required that a large number of biological data as well as a wide variety of bioinformatics tools should be publicly accessible in the cloud and delivered as services through the Internet. However, it should be also noted that biology is in its infancy (compared with other disciplines, e.g., physics) and many theoretical problems in biology are under the surface, albeit a huge volume of biological data are available now. To address theoretical problems and uncover fundamental theories in biology, it often involves hypothesis formulation, experiment design, data generation/collection, tool development (for data analysis), and knowledge formalization, which can be helpful to recognize what data and tools should be placed into the cloud.

#### Big data transfer

Transferring vast amounts of biological data to the cloud is a significant bottleneck in cloud computing [6]; it is not unusual at present to physically ship hard drives to the cloud center (http://aws.amazon.com/importexport). Currently, a promising solution is to integrate innovative transferring technologies into cloud computing. One example is the cloud-based EasyGenomics (http://www.genomics.cn/en/news/show_news?nid=99014), released by BGI (Beijing Genomics Institute), that achieves high-speed genomic data delivery by Aspera’s fasp™ high-speed file transfer technology (which dramatically speeds file transfers over the Web and outperforms conventional technologies such as FTP and HTTP; http://www.asperasoft.com/en/technology/fasp_overview_1/fasp_technology_overview_1). In June 2012, BGI succeeded in transferring genomic data across the Pacific Ocean at a sustained rate of almost 10 Gigabits per second (http://www.bio-itworld.com/news/06/29/12/Data-tsunami-BGI-transfers-genomic-data-Pacific-10-gigabits-second.html), demonstrating that high-speed transfer technologies (such as Aspera’s fasp) are capable of dealing with big data transfers over the Web. Aside from high-speed transfer technologies, there are other technologies that can also aid big data transfer, such as data compression [35,36] and Peer-to-Peer (P2P) data distribution [37,38].

#### A cloud-based lightweight programming environment

To automate data analysis, bioinformatic tasks are often implemented as pipelines by linking the output of one tool with the input of another. To perform large-scale data analysis and aid in the development of corresponding bioinformatic pipelines, a cloud-based lightweight programming environment is needed, which allows the swift development of customized pipelines from a large pool of tools and enables automated and configurable analysis on the cloud. Currently, the cloud-based programming paradigm adopted in the bioinformatics community is Hadoop [11,16,17,20,27], in which computation-consuming and data-intensive analyses are primarily solved by distributing tasks over multiple nodes (see tutorials at http://wiki.apache.org/hadoop). However, substantial computational skills are still required for developing cloud-based pipelines in Hadoop and its programming environment is not lightweight for most biologists or people with no or limited programming experience. Ideally, this would be a lightweight programming environment that does not require extensive coding by keyboard. Rather, it would be done easily by mouse, viz., “drag+drop”. Such an environment also features remote access to diverse types of resources based on utility-supplied cloud computing, consisting well with the potential trend of e-Science [39] (that is, scientific research in many disciplines is carried out via the internet). Furthermore, when building such an environment, attention should be paid to setting up a system of standards for data exchanges among different software tools [40], which can in return pave the way for realizing the full potential of lightweight programming environment.

#### Open bioinformatics clouds

Incentivized by the potential big profits to be made on a *pay-as-you-go* basis, there are multiple cloud providers and it is predicted that in the following years there will be more providers, building industrial or academic, private or public clouds. Currently, the largest provider is Amazon, which provides commercial clouds for processing big data. Additionally, Google also provides a cloud platform (https://cloud.google.com) that allows users to develop and host web applications, and store and analyze data. However, commercial clouds are currently not yet able to provide ample data and software for bioinformatics analysis. Moreover, it is very difficult for commercial clouds to keep pace with the emerging needs from academic research, consequently requiring specific clouds for bioinformatics studies. It goes without saying that open access and public availability of data and software are of great significance to science [41]. When data and software are all in the cloud, keeping the cloud open and publicly available to the scientific community is essential for bioinformatics research [42]. Therefore, it is most likely that future efforts should be devoted to building open bioinformatics clouds and providing public access to the scientific community. The potential resulting benefits of such bioinformatics clouds include easing large-scale data integration, enabling repeatable and reproducible analyses, maximizing the scope for sharing, and harnessing collective intelligence for knowledge discovery. With the presence of numerous bioinformatics clouds, interoperability and standardization between clouds will become important issues [43,44].

## Conclusions

Here we reviewed extant cloud-based resources in bioinformatics and classified them into DaaS, SaaS, PaaS, and IaaS. Since cloud computing bears great promise in effectively addressing big data storage and analysis, future efforts in building bioinformatics clouds involve developing a large variety of services from data storage, data acquisition, to data analysis, accordingly making utility-supplied cloud computing delivered over the Internet. In the era of big data, bioinformatics clouds should integrate both data and software tools, equip with high-speed transfer technologies and other related technologies in aid of big data transfer, provide a lightweight programming environment to help people develop customized pipelines for data analysis, and most important, be open and publicly accessible to the whole scientific community.

## Abbreviations

DaaS: Data as a service; SaaS: Software as a service; PaaS: Platform as a service; IaaS: Infrastructure as a service; API: Application programming interface; AWS: Amazon web service; VM: Virtual machine.

## Competing interests

The authors declare that they have no competing interests.

## Authors’ contributions

LD drafted the manuscript. XG, JX, and YG revised the manuscript critically for important intellectual content. ZZ conceived of the study, supervised the project, and revised the manuscript. All authors read and approved the final manuscript.

## Reviewers’ comments

Reviewer 1: Dr. Frank Eisenhaber (Bioinformatic Institute, Singapore)

The report by Dai et al. is a timely, well structured review of the opportunities associated with cloud computing in the bioinformatics domain and provides insight both into the existing state of the art and near-future possibilities. As such, it appears useful for the community.

At the same time, nothing is said to which extent these development have already contributed to new biological insight. Do the authors have examples for this?

*Authors’ response*: *Yes, Galaxy is a case in point; it is widely used for a wide range of bioinformatics analyses and cited by more than 300 times.*

There is the danger that the excitement with large data and with making it available shadows the actual reason why this data is recorded. Many of these so-called high impact, recently published OMICS papers are actually a boring reading with no new idea (even at the methodical level) and no new biology discovered. At the end, it is about biomolecular mechanisms that need to be known.

*Authors’ response*: *We fully agree. To reflect this point more clearly, we expanded our description accordingly. Albeit currently we have high-throughput sequencers and large-scale data, biology is likened to the 16*^*th*^*century physics in its state of development, lacking fundamental rules and theories, and many theoretical problems in biology are still under the surface. To address theoretical problems and uncover fundamental theories in biology, it often involves hypotheses formulation, experiment design, data generation/collection, tool development (for data analysis), and knowledge formalization, which can be helpful to recognize what data and tools should be placed into the cloud.*

The large data stream today is, to a great extent, due to the infantile methodology of measuring sequences and expression profiles. The TBs and PBs of sequencing data can be condensed to GBs with post-experimental processing and it are these GBs that are the actual object of scientific analysis. The human genome sequence per individual is just a few GB and, most likely and hopefully:-), it will not grow much in the future. Similarly, thousands of individual genomes might be stored as variations of a reference genome and this will, most likely, cost less than a GB per genome. Expression profiles are actually arrays of genomic locations and occurrence numbers and thus, are also in the GB range. Thus, the shift to big data science and the emphasis on new IT developments instead of doing the actual life science research might drift a considerable part of the community away into non-relevant efforts. To remember a historical example: There was a great public, large data effort with creating astronomic maps for solving the longitudinal problem in the 17th century Britain; yet, John Harrison solved it by constructing a clock.

*Authors’ response*: *Thank you for your excellent points, which we fully agree. As an interdisciplinary field, bioinformatics is influenced by any advance in IT or biology. Take Web technology as an example, in retrospect, Web 1.0 was “read-only” (50K average band width, ABW), Web 2.0 is “read-write” (1M ABW), and Web 3.0 promises to have the attributes of “read-write-execute” with greater ABW*[40]*, as cloud computing delivers all kinds of computer resources* via *the internet which to some extent aids the furtherance of Web 3.0. The rapid advancement of information technology (*e.g.*, Web technology) brings a lot to bioinformatics, including data analysis, data storage, and data sharing. Thus, it is likely inevitable that there will be a shift to “big” data science based on utility-supplied cloud computing, which is consistent with the potential trend of e-Science*[39]*(that is, scientific research in many disciplines is carried out* via *the internet). At the same time, we completely agree that “big” data to some degree result from lacks of fundamental theories; these theories can guide us in developing methods and formulating hypotheses and then know what data should be stored. Our perspectives are that in the future more and more computer scientists will help biologists build a light-weighted programming environment for biological data analysis and collaborate with biologists to conduct actual life science research. We clarified the corresponding description and added a related citation (Ref*[39]*) in the revised version.*

Reviewer 2: Dr. Igor Zhulin (University of Tennessee, United States of America)

The review summarizes advantages of using cloud computing for “big data” storage and analysis issues in bioinformatics. In general, it does a fair job on this front. However, disadvantages of clouds are not discussed in this review at all. For example, time-critical calculations, complex tasks that require data management (load balancing, fault tolerance issues, etc.) will not do well on clouds that lack the edge of advanced HPC architectures.

*Authors’ response*: *Thanks for your valuable comments. We accepted your comments and added some description in the main text. Hadoop (http://hadoop.apache.org) features two key modules—MapReduce and Hadoop Distributed File System (HDFS). MapReduce divides a computational program into many small sub-problems and distributes them on multiple computer nodes, and HDFS provides a distributed file system that stores data on these nodes. Hadoop and its associated software are designed to handle load balancing among multiple nodes and to detect node failures that can be automatically re-executed on any node. Therefore, Hadoop is capable of performing time-critical calculations by distributing tasks and large datasets over multiple computer nodes, supporting big data scaling, and enabling fault-tolerant parallelized analysis.*

Reviewer 3: Prof. Sandor Pongor (International Centre for Genetic Engineering and Biotechnology, Italy)

I am not an expert of cloud computing but I am am very curious to see the potentials of this technology for bioinformatics. I found the paper clearly written, concise and well structured - it gives good introduction to this field, that readers of this journal will appreciate in my opinion. What I kind of miss is the user's perspective. When shall a student or a user of bioinformatics think about a cloud solution? Where to get introductory texts? On a more practical side: Teaching the bioinformatics of big data (high throughput) seems to be easy by putting a virtual server on the cloud … Where are the could based courses? These are simple questions and the authors may want to dedicate a little space to dealing with them.

*Authors’ response: Thank you for your thoughtful and excellent comments. “When shall a student or a user of bioinformatics think about a cloud solution”, is highly dependent on the user’s need and the computer resources the user has. Taking SaaS as an example, if a computational task is very time-consuming and also this task can be divided into many small sub-tasks, it might be better to put this task into the cloud and to run it as SaaS. The cloud just mentioned can be offered by a cloud provider (*e.g.*, Amazon), or be in-house, self-made which makes full use of multiple computers available in one lab/institution. The relevant introductory texts are available athttp://wiki.apache.org/hadoop, which provides general information and tutorials on how to build a Hadoop-based cloud and includes several practical examples. There is an online course—“Cloud Application Development” athttp://my.ss.sysu.edu.cn/cloud/, which also contains other related resources and references. In addition, Galaxy (https://main.g2.bx.psu.edu) is capable of performing interactive data analyses, which can serve as an E-learning platform for students.*

## References

[B1] Schatz MC Langmead B Salzberg SL Cloud computing and the DNA data race Nat Biotechnol 2010 28 7 691 693 10.1038/nbt0710-691 20622843 PMC2904649

[B2] Eisenstein M Oxford Nanopore announcement sets sequencing sector abuzz Nat Biotechnol 2012 30 4 295 296 10.1038/nbt0412-295 22491260

[B3] Schadt EE Linderman MD Sorenson J Lee L Nolan GP Cloud and heterogeneous computing solutions exist today for the emerging big data problems in biology Nat Rev Genet 2011 12 3 224 21301474 10.1038/nrg2857-c2

[B4] Schadt EE Linderman MD Sorenson J Lee L Nolan GP Computational solutions to large-scale data management and analysis Nat Rev Genet 2010 11 9 647 657 10.1038/nrg2857 20717155 PMC3124937

[B5] Grossman RL White KP A vision for a biomedical cloud J Intern Med 2012 271 2 122 130 10.1111/j.1365-2796.2011.02491.x 22142244 PMC6338328

[B6] Armbrust M Fox A Griffith R Joseph AD Katz RH Konwinski A Lee G Patterson DA Rabkin A Stoica I Above the Clouds: A Berkeley View of Cloud Computing 2009 Berkeley: EECS Department, University of California

[B7] Garfinkel SL Architects of the Information Society: Thirty-Five Years of the Laboratory for Computer Science at MIT 1999 Cambridge, MA: The MIT Press

[B8] Buyya R Yeo CS Venugopal S Broberg J Brandic I Cloud computing and emerging IT platforms: vision, hype, and reality for delivering computing as the 5th utility Future Gener Comp Sy 2009 25 6 599 616 10.1016/j.future.2008.12.001

[B9] Dudley JT Butte AJ In silico research in the era of cloud computing Nat Biotechnol 2010 28 11 1181 1185 10.1038/nbt1110-1181 21057489 PMC3755123

[B10] Stein LD The case for cloud computing in genome informatics Genome Biol 2010 11 5 207 10.1186/gb-2010-11-5-207 20441614 PMC2898083

[B11] Taylor RC An overview of the Hadoop/MapReduce/HBase framework and its current applications in bioinformatics BMC Bioinformatics 2010 11 Suppl 12 S1 10.1186/1471-2105-11-S12-S1 21210976 PMC3040523

[B12] Stanoevska-Slabeva K Wozniak T Stanoevska K, Wozniak T, Ristol S Cloud Basics - An Introduction to Cloud Computing Grid and Cloud Computing: Business Perspective on Technology and Applications 2010 Berlin: Springer 47 61

[B13] Truong HL Dustdar S On Analyzing and Specifying Concerns for Data as a Service 2009 Ieee Asia-Pacific Services Computing Conference (Apscc 2009) 2009 83 90

[B14] DaaS: The New Information Goldmine http://online.wsj.com/article/SB125071202052143965.html

[B15] Fusaro VA Patil P Gafni E Wall DP Tonellato PJ Biomedical cloud computing with Amazon Web Services PLoS Comput Biol 2011 7 8 e1002147 10.1371/journal.pcbi.1002147 21901085 PMC3161908

[B16] Nguyen T Shi W Ruden D CloudAligner: a fast and full-featured MapReduce based tool for sequence mapping BMC Res Notes 2011 4 171 10.1186/1756-0500-4-171 21645377 PMC3127959

[B17] Schatz MC CloudBurst: highly sensitive read mapping with MapReduce Bioinformatics 2009 25 11 1363 1369 10.1093/bioinformatics/btp236 19357099 PMC2682523

[B18] Langmead B Schatz MC Lin J Pop M Salzberg SL Searching for SNPs with cloud computing Genome Biol 2009 10 11 R134 10.1186/gb-2009-10-11-r134 19930550 PMC3091327

[B19] Matsunaga A Tsugawa M Fortes J Combining MapReduce and Virtualization on Distributed Resources for Bioinformatics Applications Fourth IEEE International Conference on eScience 2008 222 229

[B20] Hong D Rhie A Park SS Lee J Ju YS Kim S Yu SB Bleazard T Park HS Rhee H FX: an RNA-Seq analysis tool on the cloud Bioinformatics 2012 28 5 721 723 10.1093/bioinformatics/bts023 22257667

[B21] Langmead B Hansen KD Leek JT Cloud-scale RNA-sequencing differential expression analysis with Myrna Genome Biol 2010 11 8 R83 10.1186/gb-2010-11-8-r83 20701754 PMC2945785

[B22] Zhang L Gu S Liu Y Wang B Azuaje F Gene set analysis in the cloud Bioinformatics 2012 28 2 294 295 10.1093/bioinformatics/btr630 22084254

[B23] Wall DP Kudtarkar P Fusaro VA Pivovarov R Patil P Tonellato PJ Cloud computing for comparative genomics BMC Bioinformatics 2010 11 259 10.1186/1471-2105-11-259 20482786 PMC3098063

[B24] Feng X Grossman R Stein L PeakRanger: a cloud-enabled peak caller for ChIP-seq data BMC Bioinformatics 2011 12 139 10.1186/1471-2105-12-139 21554709 PMC3103446

[B25] Habegger L Balasubramanian S Chen DZ Khurana E Sboner A Harmanci A Rozowsky J Clarke D Snyder M Gerstein M VAT: a computational framework to functionally annotate variants in personal genomes within a cloud-computing environment Bioinformatics 2012 Epub ahead of print 10.1093/bioinformatics/bts368 PMC3426844 22743228

[B26] Wang Z Wang Y Tan KL Wong L Agrawal D eCEO: an efficient Cloud Epistasis cOmputing model in genome-wide association study Bioinformatics 2011 27 8 1045 1051 10.1093/bioinformatics/btr091 21367868

[B27] Jourdren L Bernard M Dillies M-A Le Crom S Eoulsan: a cloud computing-based framework facilitating high throughput sequencing analyses Bioinformatics 2012 published online April 5, 20122010.1093/bioinformatics/bts2165 10.1093/bioinformatics/bts165 22492314

[B28] Afgan E Baker D Coraor N Goto H Paul IM Makova KD Nekrutenko A Taylor J Harnessing cloud computing with Galaxy Cloud Nat Biotechnol 2011 29 11 972 974 10.1038/nbt.2028 22068528 PMC3868438

[B29] Afgan E Baker D Coraor N Chapman B Nekrutenko A Taylor J Galaxy CloudMan: delivering cloud compute clusters BMC Bioinformatics 2010 11 Suppl 12 S4 10.1186/1471-2105-11-S12-S4 21210983 PMC3040530

[B30] Krampis K Booth T Chapman B Tiwari B Bicak M Field D Nelson K Cloud BioLinux: pre-configured and on-demand bioinformatics computing for the genomics community BMC Bioinformatics 2012 13 1 42 10.1186/1471-2105-13-42 22429538 PMC3372431

[B31] Angiuoli SV Matalka M Gussman A Galens K Vangala M Riley DR Arze C White JR White O Fricke WF CloVR: a virtual machine for automated and portable sequence analysis from the desktop using cloud computing BMC Bioinformatics 2011 12 356 10.1186/1471-2105-12-356 21878105 PMC3228541

[B32] Dudley JT Pouliot Y Chen R Morgan AA Butte AJ Translational bioinformatics in the cloud: an affordable alternative Genome Med 2010 2 8 51 10.1186/gm172 20691073 PMC2945008

[B33] Zhang Z Bajic VB Yu J Cheung K-H Townsend JP Mahdavi MA Data Integration in Bioinformatics: Current Efforts and Challenges Bioinformatics - Trends and Methodologies 2011 Rijeka, Croatia: InTech - Open Access Publisher

[B34] Fox A Cloud computing-what's in it for me as a scientist? Science 2011 331 6016 406 407 10.1126/science.1198981 21273473

[B35] Deorowicz S Grabowski S Compression of DNA sequence reads in FASTQ format Bioinformatics 2011 27 6 860 862 10.1093/bioinformatics/btr014 21252073

[B36] Cox AJ Bauer MJ Jakobi T Rosone G Large-scale compression of genomic sequence databases with the Burrows-Wheeler transform Bioinformatics 2012 28 11 1415 1419 10.1093/bioinformatics/bts173 22556365

[B37] Langille MGI Eisen JA BioTorrents: a file sharing service for scientific data PLoS One 2010 5 4 e10071 10.1371/journal.pone.0010071 20418944 PMC2854681

[B38] Sangket U Phongdara A Chotigeat W Nathan D Kim WY Bhak J Ngamphiw C Tongsima S Khan AM Lin H Automatic synchronization and distribution of biological databases and software over low-bandwidth networks among developing countries Bioinformatics 2008 24 2 299 301 10.1093/bioinformatics/btm570 18037613

[B39] Bishop M e-Science Brief Bioinform 2003 4 3 208 209 10.1093/bib/4.3.208 14582515

[B40] Zhang Z Cheung KH Townsend JP Bringing Web 2.0 to bioinformatics Brief Bioinform 2009 10 1 1 10 18842678 10.1093/bib/bbn041 PMC2638627

[B41] Marx V My data are your data Nat Biotechnol 2012 30 6 509 511 10.1038/nbt.2243 22678394

[B42] Rosenthal A Mork P Li MH Stanford J Koester D Reynolds P Cloud computing: a new business paradigm for biomedical information sharing J Biomed Inform 2010 43 2 342 353 10.1016/j.jbi.2009.08.014 19715773

[B43] Dillon T Wu C Chang E Cloud Computing: Issues and Challenges Int Con Adv Info Net 2011 27 33

[B44] Parameswaran AV Chaddha A Cloud interoperability and standardization SETLabs Briefings 2009 7 7 19 26

